# Nasogastric tube feeding under physical restraint: comprehensive audit and case series across in-patient mental health units in England

**DOI:** 10.1192/bjb.2023.30

**Published:** 2023-12

**Authors:** Sarah J. Fuller, Jacinta Tan, Huw De Costa, Dasha Nicholls

**Affiliations:** 1 Imperial College London, UK; 2 East London NHS Foundation Trust, UK; 3 Oxford Health NHS Foundation Trust, UK; 4 University of Oxford, UK

**Keywords:** Nasogastric tube feeding, restraint, restrictive practices, audit, eating disorders

## Abstract

**Aims and method:**

To identify the clinical characteristics of patients receiving nasogastric tube (NGT) feeding under physical restraint. Clinicians participated via professional networks and subsequent telephone contact. In addition to completing a survey, participants were invited to submit up to ten case studies.

**Results:**

The survey response rate from in-patient units was 100% and 143 case studies were submitted. An estimated 622 patients received NGT feeding under restraint in England in 2020–2021. The most common diagnosis was anorexia nervosa (68.5–75.7%), with depression, anxiety and autism spectrum disorder the most frequent comorbidities. Patients receiving the intervention ranged from 11 to 60 years in age (mean 19.02 years). There was wide variation in duration of use, from once to daily for 312 weeks (mode 1 week; mean 29.1 weeks, s.d. = 50.8 weeks).

**Clinical implications:**

NGT feeding under restraint is not uncommon in England, with variation in implementation. Further research is needed to understand how the high comorbidity and complexity contribute to initiation and termination of the intervention.

When patients are admitted to mental health in-patient units for restrictive eating disorders/disordered eating one of the primary goals of treatment is medical stabilisation and promotion of physical health.^1^ This can be achieved in a number of ways: support to eat an oral diet, meal-replacement supplement drinks or, if these are not possible, nasogastric tube (NGT) feeding.^2^ It is not uncommon for patients to accept NGT feeding as the method of nutritional intake that induces the least guilt.^3^ However, if a patient is unable to accept oral intake or NGT feeding on a voluntary basis, NGT feeding under physical restraint may be required.

Little is known about the frequency with which NGT feeding under restraint is used nor the characteristics of patients likely to receive this intervention. It is usually the case that NGT feeding under restraint is justified as a life-saving option in situations of severe physical compromise. However, anecdotally this may escalate conflict between the treating team and the patient or entrench refusal of intake, resulting in repeated use. Furthermore, clinical experience tells is that NGT feeding under physical restraint may also become a longer-term intervention used to fully restore physical health.

Recent developments have been aimed at improving practice and increasing the evidence base. Dietetic guidelines regarding how to implement NGT feeding in the least restrictive manner^4,5^ and guidance on legal and ethical principles for when this intervention is needed outside specialist mental health units, such as on acute paediatric wards,^6^ have been published. Furthermore, some qualitative research has explored the patient experience^3^ and that of nursing assistants when this intervention is needed.^7^ However, there is no published research about the prevalence of this practice, and evidence to guide best practice in managing the intervention from a medical and psychological perspective, including the impact on patients, families and staff, is lacking.

## Aims

To estimate how many patients in mental health in-patient units in England received NGT feeding under physical restraint during a 12-month period (June 2020 to May 2021), the patient demographics and clinical characteristics, and for how long the intervention was delivered.

## Method

### Design

We conducted an online survey open to clinicians working in in-patient mental health settings in England.

NHS England provided a list of all national health service (NHS) and independent sector child and adolescent mental health units in England admitting NHS patients, together with a list of all NHS and independent sector adult specialist eating disorder units (SEDUs). In addition, non-SEDU adult mental health units in two NHS regions (Thames Valley and Wessex; East Midlands) were asked for data to ensure that focusing solely on SEDUs for the adult population was likely to capture most, if not all, cases. Out of 91 adult non-SEDU wards (including general adult, psychiatric intensive care, low secure, medium secure, intellectual disability, deaf, personality disorder, rehabilitation and stepdown wards), only one specialist personality disorder ward was able to implement NGT feeding under restraint. The team concluded that including non-SEDU adult mental health units across all of England was not justified for the likely yield of additional cases.

Conduct of the study was overseen by a study steering group, consisting of the research team, expert clinicians (including an in-patient eating disorder consultant psychiatrist, a medical ethicist and senior nurses), a person with lived experience and the carer of someone who had experienced this intervention. Meetings were held to guide the development of the project: the research aims, audit questions, case study requirements and interpretation of results.

### Survey distribution

Responses to the survey were requested from psychiatrists, hospital directors, nurse managers or senior multidisciplinary team (MDT) clinicians. Respondents were invited to submit up to ten cases studies in which the patient had received NGT feeding under physical restraint.

The survey and case studies were hosted by the online platform Qualtrics and links were sent to units via faculties or networks within the Royal College of Psychiatrists (RCPsych) (Faculty of Eating Disorders Psychiatry, Quality Network for In-patient Child and Adolescent Mental Health Services (CAMHS), Quality Network for Eating Disorders, Quality Network for Psychiatric Intensive Care Units) and via a post on the British Eating Disorders Society (BrEDS) platform.

The 12-month reporting period was June 2020–May 2021. Units that had not responded to the online survey after 3 months were individually telephoned to encourage participation. Units submitting duplicate submissions (*n* = 11) and incomplete submissions (*n* = 5) were contacted via phone to discuss and clarify their responses. Responses from outside of England were excluded, as were responses from acute paediatric wards (*n* = 2). For the case series, incomplete submissions were deleted (*n* = 17) and responses from outside of England (identified from IP address location) (*n* = 3) were also excluded.

### Ethics and information governance

East London NHS Foundation Trust Research and Development Department approved this study as a clinical audit, exempt from ethics approval.

Participants consented to this research project by agreeing to an electronic statement at the start of data gathering. All data received for the case series were fully anonymised and personal patient data were never collected. Participating clinicians submitting the data remained anonymous but the site they worked at was identified in order to calculate the response rate.

## Results

### Demographics

A 100% response rate was achieved from all CAMHS units (including CAMHS SEDUs) (*n* = 109) and adult SEDUs (*n* = 35) in England.

In total, 143 anonymised case studies were submitted for analysis from 62 mental health units.

#### How many patients required NGT feeding under physical restraint in England during the reporting period?

A reported 622 patients required NGT feeding under physical restraint between June 2020 and May 2021. In total, 78.1% (*n* = 486) were in CAMHS units and 21.9% (*n* = 136) in adult SEDUs. Within CAMHS services, 64% (*n* = 311) were in CAMHS SEDUs and 36% (*n* = 175) in CAMHS non-SEDUs.

#### Characteristics of patients who received NGT feeding under physical restraint

##### Primary diagnosis/presentation

Across the audit and case series, the predominant diagnosis was anorexia nervosa, with a frequency of 68.5–75.7%, depending on the type of unit. Food refusal in the context of emotional dysregulation/emerging personality disorder was the second most common presentation, with a frequency ranging from 11.2 to 17.1% by unit type (Table 1).

**Table 1:** Primary diagnosis/presentation of patients in the audit and case series

Primary diagnosis	CAMHS non-SEDU (*n* = 175)	CAMHS SEDU (*n* = 311)	Adult SEDU (*n* = 136)	Case series (*n* = 143)
Anorexia nervosa	126 (72.0%)	236 (75.9%)	103 (75.7%)	98 (68.5%)
Bulimia nervosa	−	10 (3.2%)	4 (2.9%)	1 (0.7%)
Atypical eating disorder (EDNOS/OSFED)	14 (8.0%)	28 (9.0%)	9 (6.7%)	13 (9.1%)
Avoidant restrictive food intake disorder	−	2 (0.6%)	−	5 (3.5%)
Other: food refusal in the absence of eating disorder psychopathology i.e. Emotional dysregulation, EUPD/BPD/PD	30 (17.1%)	33 (10.6%)	19 (14.0%)	16 (11.2%)
Other: anxiety	−	−	−	2 (1.4%)
Other: depression	−	−	−	1 (0.7%)
Other: catatonic state	2 (1.1%)	−	1 (0.7%)	−
Other: psychosis	2 (1.1%)	−	−	−
Other: pervasive arousal withdrawal syndrome	−	2 (0.6%)	−	−
Other: obsessive–compulsive disorder	1 (0.6%)	−	−	1 (0.7%)
Other: disordered eating	−	−	−	1 (0.7%)
Other: gender dysphoria	−	−	−	1 (0.7%)
Other: suicidal ideation	−	−	−	1 (0.7%)
Other: food avoidance emotional disorder	−	−	−	1 (0.7%)
Other: unknown	−	−	−	2 (1.4%)

CAMHS, child and adolescent mental health services; SEDU, specialist eating disorder unit; EDNOS, eating disorder not otherwise specified; OSFED, other specified feeding or eating disorder; EUPD, emotionally unstable personality disorder; BPD, borderline personality disorder; PD, personality disorder.

##### Case series

**Fig. 1:**
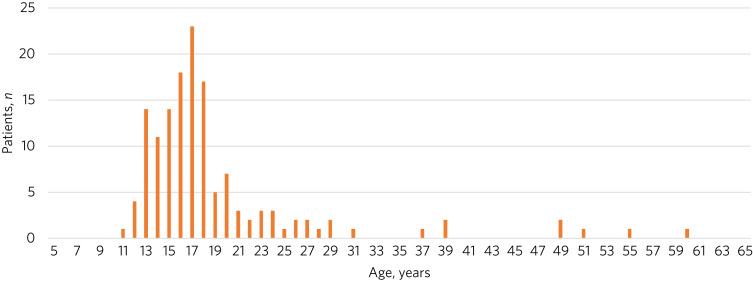
Age distribution of patients in the submitted cases studies (*n* = 143).

For the 143 cases studies submitted, the mean age of patients was 19.02 years (s.d. = 7.9) (Fig. 1). In total, 123 (86%) of the patients were White British, 6 (4.2%) ‘other White’, 6 (4.2%) ‘mixed race’ and 8 (5.6%) were other specified ethnicities (Chinese, Black, Asian); 111 (77.6%) of the patients were cis-females, 2 (1.4%) cis-males and 30 (21%) were reported as a gender different from that they were born with, of whom 24 (16.9%) identified as transgender, 5 (3.5%) as non-binary and 1 (0.7%) was unknown.

Within the case series, secondary diagnoses were reported both pre-admission and during admission. Of the patients described, 84.6% (*n* = 121) had at least one secondary diagnosis on admission. The most common secondary diagnoses were depression (*n* = 60, 42%) and anxiety (*n* = 60, 42%) and these were predominantly diagnosed prior to the admission. Furthermore, during the admissions reported, there were high rates of autism spectrum disorder (*n* = 47, 32.9%) and emotionally unstable personality disorder (EUPD) (*n* = 40, 28%).

#### How long was the intervention needed for?

**Fig. 2:**
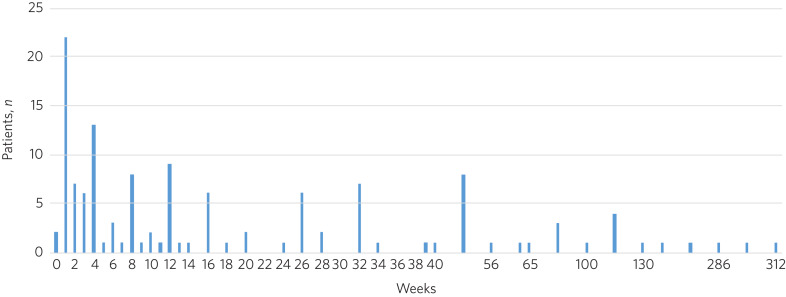
Estimated duration of nasogastric tube feeding under restraint reported in the submitted cases studies (*n* = 143).

The length of time NGT feeding under restraint was delivered ranged from a single feed to 312 weeks, the mean was 29.1 weeks (s.d. = 50.5) and the mode was 1 week (Fig. 2); 17 patients had been fed under restraint for over 1 year.

## Discussion

We report the first national survey and case series of patients who have received NGT feeding under restraint in English mental health wards. For the under-18 patient group, data were collected from general adolescent mental health units as well as specialist eating disorder in-patient units (SEDUs), and for the 18 years and over (adult) patient group, data were collected only from SEDUs. The use of NGT feeding in adult non-SEDUs is extremely rare.

### Clinical characteristics

A total of 622 patients are reported to have received this intervention over a 1-year period in the mental health settings surveyed. The primary clinical population in which this intervention was used was patients with anorexia nervosa, with other eating disorders and ‘emotional dysregulation’ making up the majority of other cases. Emotional dysregulation is a transdiagnostic term often, but not exclusively, associated with emerging or diagnosed personality disorder. However, there were also patients receiving this intervention who had a primary diagnosis of obsessive–compulsive disorder, psychosis, pervasive arousal withdrawal syndrome, anxiety, depression, suicidal ideation and gender dysphoria. This reflects that malnutrition and physical decompensation is seen in a range of mental disorders and not solely driven by weight or shape concerns, as seen in those with eating disorders.^8^ This finding is important from a dietetic perspective as current guidance on how to modify standard practice to deliver nutrition to those who require NGT feeding under restraint is applicable only to those with anorexia nervosa^4,5^ and should be widened to those who have a mental health condition and severe malnutrition as a result of this.

Regarding the reported secondary diagnoses, research suggests that our findings are mirrored in the wider eating disorder population, with high rates of anxiety,^9^ depression,^9^ autism spectrum disorder^10^ and emotional dysregulation. However, this is the first paper to report that these diagnoses and presentations may indicate that a patient is more likely to require NGT feeding under restraint. Understanding this, clinicians may be able to adapt their formulations and treatment plans accordingly, rather than taking a ‘one size fits all’ approach to the intervention.

### Demographics

The mean age of those requiring NGT feeding under restraint was 19 years and the mode was 17 years. In the 143 case studies, the age distribution was similar to that seen in clinical service presentations, i.e. a mean age of 19.02 years (s.d. = 7.9), with eight patients over the age of 35. Furthermore, the distribution of age (Fig. 1) indicates that this intervention is most commonly used in children and young people under the age of 18 years. This may be because of the different perception of risk that clinicians have with younger patients or it may be related to factors such as likelihood of escalating conflict with treating teams. Although there are clear guidelines regarding when a patient is becoming medically unstable and requires either review by an experienced acute medical physician or admission to a medical hospital,^11^ this guidance does not clarify when a patient's life is at risk and a restrictive intervention such as NGT feeding under restraint is necessary.

In our sample, the predominant gender, as expected, was cis-female (*n* = 111, 77.6%). Interestingly, 20.3% of patients identified as transgender (*n* = 29) or non-binary (*n* = 3). This may reflect bias in clinician recall,^12^ as clinicians were asked to report case studies rather than undertake a systematic retrospective case note review. However, it is important to acknowledge that when a patient's eating disorder is underpinned by gender dysphoria there is currently little evidence of how to best support them aside from providing gender-affirmative care,^13,14^ suggesting that gender awareness and treating gender identity disclosure as routine are important.^15^ Research suggests that this group of patients experience high levels of bullying and trauma,^16,17^ there is a higher lifetime risk of eating disorders in those who are identified as female at birth but subsequently identify as male^18^ and that there is a significant co-occurrence of gender identity dysphoria and autism.^19,20^ Therefore, it is especially important to consider the impact of NGT feeding under restraint in this group of patients as they may be at higher risk of prolonged restraints and risk of reliving trauma.

### Duration of NGT feeding

The mean number of weeks NGT feeding under restraint was used for was 29.1 weeks. There is research to suggest that individuals with anorexia nervosa who receive NGT feeding during their in-patient treatment have a longer length of stay.^21^ However, our finding implies that NGT feeding under restraint was used beyond the point of medical stabilisation (typically achieved within around 10 days) and became a core part of ongoing treatment for some patients. This could reflect it being the only way some patients with severe and enduring eating disorders can accept weight gain, or indicate patients with complex presentations or those who are unable to maintain their safety outside of mental health in-patient units.

### Implications for clinical practice and further research

By capturing detailed information on a national sample of patients fed by NGT under restraint, our study allows a clinical picture to be formed of those patients most likely to receive this intervention. Importantly, our study raises questions about the legal and clinical basis for the practice, and whether changes to practice might influence the frequency with which this potentially traumatic intervention is utilised.

Our data also suggest that some patients may become dependent on this method of nutritional intake, requiring the intervention for months or years. This suggests that clinicians need to be very careful to consider the ethical and legal justification for the use of such coercive measures, which involve physical restraint, and to be vigilant for ‘a slippery slope’ when something morphs from being purely life-saving in best interests to a default means of maintaining weight gain in the face of continued conflict or difficulty in providing sufficient nutrition. What is the likely impact of discontinuing the intervention, how do they shift the emphasis away from coercive feeding to more cooperative nutrition, and how do they weigh up the risks of some weight loss while trying to move back to voluntary intake against the risks of continuing? A nuanced understanding of the decision-making processes involved, the factors that contribute to prolonged feeding under restraint and effective strategies for discontinuing feeding under restraint is needed to guide clinical care.

Further research is needed to fully understand these findings. In particular, understanding why some patients require prolonged NGT feeding under restraint and whether those with comorbid presentations require different care planning approaches to prevent the need for this intervention. Research is also needed to understand the process by which patients come to be fed under restraint and the role of the clinician in patient-centred discussions and shared decision-making and to understand how clinicians can plan an exit strategy before starting this intervention: such planning involves considering whether the purpose of the intervention is time-limited medical stabilisation or full weight restoration. In addition, research needs to elucidate the impact of the intervention on patients, families and staff, how to optimise delivery of the intervention and factors that may reduce the frequency or duration of the intervention.

### Strengths and weaknesses

The response rate was 100% from units likely to deliver this intervention, meaning the data accurately reflect national prevalence and it is hoped that subsequent audits and improvements in practice will show a reduced incidence of NGT feeding under restraint.

There are a few limitations regarding the audit. As this is a self-reported survey relying on recall of a 12-month period, there may be clinician bias;^12^ also, some patients may be counted twice if they transferred units during the reporting period. Furthermore, when recalling patients’ secondary diagnoses, reporting allowed multiple secondary or suspected diagnoses; therefore, it was not possible to generate a percentage or proportion of cases, as with the primary diagnosis. However, we are able to say that patients are more likely to have comorbid anxiety, depression, autism spectrum disorder and EUPD. Finally, we are aware that NGT feeding under physical restraint occurs in settings other than in-patient mental health units, such as acute medical or paediatric wards, which were excluded using the current methodology.

It is important to note that although general adolescent in-patient units generally have expertise in using NGT feeding under restraint and in this audit would have reported its use for patients who did not have eating disorders, we did not survey general adult in-patient units so cannot comment on potential use of NGT feeding under restraint for adults without eating disorders in these settings. Therefore we are unable to comment on potential use of NGT feeding under restraint for adults without eating disorders with, for example, obsessive–compulsive disorder or emotional dysregulation with food refusal.

## About the authors

**Sarah J. Fuller** is an eating disorders clinician with East London NHS Foundation Trust and a research dietitian in the Division of Psychiatry at Imperial College London, UK. **Jacinta Tan** is a consultant child and adolescent psychiatrist with Oxford Health NHS Foundation Trust and a Senior Clinical Research Fellow in the Department of Psychiatry at the University of Oxford, UK. **Huw De Costa** is a junior doctor on the GP trainee scheme in the Division of Psychiatry at Imperial College London, UK. **Dasha Nicholls** is a Clinical Reader in Child and Adolescent Psychiatry in the Division of Psychiatry at Imperial College London, UK.
